# Healthy eating index versus alternate healthy index in relation to diabetes status and health markers in U.S. adults: NHANES 2007–2010

**DOI:** 10.1186/s12937-019-0450-6

**Published:** 2019-04-17

**Authors:** Afnan A. Al-Ibrahim, Robert T. Jackson

**Affiliations:** Department of Nutrition and Food Science, University of Maryland, 0112 Skinner Building, College Park, MD 20742 USA

**Keywords:** NHANES, HEI-2010, AHEI-2010, Diabetes Status, Health Markers

## Abstract

**Background:**

It remains to be determined whether the Alternate Healthy Eating Index 2010 (AHEI-2010) or the Healthy Eating Index 2010 (HEI-2010) is preferably recommended as means to assess dietary quality in people with type 2 diabetes (T2DM).

**Methods:**

The purpose of this study was to determine whether the AHEI-2010 provides a more accurate assessment of dietary quality than the HEI-2010 in relation to diabetes status, while controlling for health markers, sociodemographic and lifestyle factors. The 2007–2010 National Health and Nutrition Examination Survey (NHANES) was used as a representative sample of U.S. adults age 20+ years (*n* = 4097). HEI-2010 and the AHEI-2010 scores were used as measures of dietary quality and were calculated using data from the first 24-h dietary recall. Health markers evaluated include anthropometrics, blood pressure, lipid and inflammatory markers, and presence of co-morbid diseases. Least Squares Means were computed to determine differences across diabetes status (nondiabetes, prediabetes, T2DM) for total and sub-component HEI-2010 and AHEI-2010 scores, and to determine differences across total HEI-2010 and AHEI-2010 quartiles for health markers. Covariate-adjusted logistic regression was used to examine the association between total HEI-2010 and AHEI-2010 scores and diabetes status.

**Results:**

Adults with T2DM showed higher HEI-2010 and AHEI-2010 scores compared to adults with prediabetes and nondiabetes but did not have better health markers. For HEI-2010 component scores, adults with T2DM had highest consumption (highest score) of total protein foods and lowest consumption (highest score) for empty calories (*p* < 0.01). For AHEI-2010 component scores, adults with T2DM had the lowest consumption (highest score) for sugar-sweetened beverages and fruit juice, sodium, and alcohol (lowest score). In addition, adults with T2DM had the highest consumption (lowest score) for red and/or processed meats (*p* < 0.01). However, neither total HEI-2010 nor AHEI-2010 scores were significantly associated with diabetes status (*p* > 0.05). Results suggest that neither index was clearly superior to the other in terms of its predictive ability in relation to T2DM.

**Conclusion:**

Neither total HEI-2010 nor AHEI-2010 scores performed better in terms of their relationship with diabetes status. However, the significant relationships between 1) diabetes status and health markers and 2) between HEI-2010 and AHEI-2010 scores and health markers suggest that diet has some influence on T2DM.

**Electronic supplementary material:**

The online version of this article (10.1186/s12937-019-0450-6) contains supplementary material, which is available to authorized users.

## Introduction

Type 2 diabetes mellitus (T2DM) is a serious clinical and public health concern in the United States [1, 2]. T2DM continues to be prevalent despite public health efforts to develop effective policies and interventions. In 2012, it is estimated that about 12.3% of U.S. adults age 20 years and older had diagnosed or undiagnosed diabetes [3]. T2DM is associated with an increased risk of serious complications including cardiovascular disease (CVD) and is a primary risk factor for coronary heart disease (CHD) [4]. It is estimated that at least 68% of people aged 65 years or older with T2DM die from CVD in the United States [5]. Moreover, a recent meta-analysis by Einarson and colleagues (2017) examined the prevalence of CVD among adults (mean age 63.6 ± 6.9 years) with T2DM during the time period between 2007 and 2017 in multiple countries, including the United States [6]. Results indicate that about 32.2% of individuals with T2DM were affected by overall CVD. CHD was found to be the most prevalent contributor of CVD mortality (about 21.2%) among individuals with T2DM [6]. Moreover, an analysis of data from the 2009–2012 National Health and Examination Survey (NHANES) found that about 37% of U.S. adults age 20 years and older (51% of those age 65 years or older) had prediabetes. People with prediabetes had high fasting plasma glucose (FPG) or hemoglobin A1c (HbA1c) levels, but these blood values were not high enough yet for a diagnosis of T2DM [3]. However, prediabetes increases the risk of developing T2DM, heart disease, and stroke in the future. Estimates from the Centers for Disease Control and Prevention (CDC) suggest that about 15–30% of people with prediabetes will develop T2DM within five years [3]. Therefore, lifestyle interventions to improve diet are an important strategy to prevent T2DM and other adverse health outcomes, and optimize long-term health [7].

The Healthy Eating Index-2010 (HEI-2010) is a measure of diet quality in relation to the 2010 Dietary Guidelines for Americans (DGA 2010) [8]. The main objective of DGA 2010 is to promote healthy eating in the general population [9]. The HEI-2010 score captures key nutrients and food groups that reflects current evidence on the dietary components that are healthful [10]. Another popular tool used to measure dietary quality is the Alternate Healthy Eating Index-2010 (AHEI-2010), which is based on evidence-based recommendations that incorporates additional components that focus on foods and nutrients to predict the risk of chronic disease [11, 12]. The most recent U.S. dietary guidelines (DGA 2015) have somewhat moved in the direction suggested by the AHEI [9]. For instance, the HEI-2015 has included added sugars and saturated fats as two separate components instead of being combined into empty calories, which is one of the components in HEI-2010 [13]. However, excessive calories from alcohol (part of empty calories) has been removed in the HEI-2015 whereas the AHEI-2010 includes alcohol as a separate component to assess dietary quality. With the exception of these minor changes, most of the HEI-2010 components are kept in HEI-2015 [13]. The AHEI-2010 provides additional food and nutrient components that are neither found in HEI-2010 nor HEI-2015. Therefore, it is useful to utilize the HEI and AHEI indices to examine their association with health or disease outcomes, such as T2DM.

The HEI-2010 and AHEI-2010 are useful tools measure adherence to dietary guidelines and evidence-based recommendations. The HEI-2010 and AHEI-2010 are similar in some aspects. For example, both indices capture consumption of fruits, vegetables, whole grains, and sodium. However, the AHEI-2010 reflects a critique of the HEI-2010 where it provides dietary recommendations that better improve health risk factors, and it has shown to more strongly predict chronic disease risk (i.e., T2DM) and mortality [11, 12]. The AHEI-2010 incorporates distinct features from the HEI-2010. For example, the AHEI-2010 pays more attention to fat quality (i.e., intakes of omega-3 fats and polyunsaturated fats), promotes intake of nuts and legumes, and considers moderate alcohol intake (Male: 0.5–2.0 drinks/day; Female: 0.5–1.5 drinks/day) as beneficial to health regardless of disease status (i.e., diabetes). In addition, the AHEI-2010 recommends to limit intake of red and processed meats and avoid added sugars (i.e., sugar-sweetened beverages and fruit juice) [12]. Both HEI-2010 and AHEI-2010 complement one another in terms of evaluating essential foods groups and nutrients. Therefore, it is useful to utilize the HEI-2010 and AHEI-2010 as tools to assess dietary quality and examine their association with health markers and diabetes status.

Several prospective studies have evaluated HEI-2010 and AHEI-2010 scores in relation to T2DM [11–18]. Results from a recent meta-analysis of 15 cohort studies, with follow-up time ranging from 5 to ≥24 years, show that diets of the highest quality (compared highest vs. lowest quintile scores), as assessed by the HEI, AHEI, and DASH, are associated with a significant risk reduction for all-cause mortality, T2DM and other chronic diseases (i.e., cardiovascular disease, cancer) (*P* < 0.05) [18]. The meta-analysis included seven reported studies (six in the United States and one in Europe) on T2DM as the main disease outcome, with age ranging from 30 to 79 years, among individuals from different ethnic groups including Caucasian (European), non-Hispanic White, African American, Hispanic, and Asian. Of these studies, the main result indicates that diets that score highly on the HEI, AHEI, and DASH are associated with a significant reduction in the risk of T2DM (22%, *P* < 0.05) [18]. In these studies, the HEI-2010 (and HEI-2005) has been evaluated among individuals with chronic disease (including T2DM) with mixed results. Some studies have shown moderate inverse associations and some showed no association with regards to the HEI-2010 and T2DM risk [12, 17]. However, the AHEI-2010 (and AHEI-2005) has demonstrated to be more strongly associated with chronic disease, including T2DM [11, 12, 14, 15, 17]. McCullough and colleagues evaluated whether or not the AHEI-2005 would predict risk reduction for chronic disease (including CVD, cancer, or nontraumatic death) more effectively than the HEI-2005 [11]. The study was conducted in the United States among females aged 30–75 years enrolled in the Nurses’ Health Study (NHS), and males aged 40–75 years participated in the Health Professional’s Follow-up Study (HPFS). The main result indicates that the AHEI-2005 was more effective in predicting chronic disease risk than the HEI-2005. The overall risk reduction with the AHEI-2005 (highest quintile compared to lowest quintile) was lower among men and women, with 11 and 3%, respectively [11]. In 2012, Chiuve and colleagues used the NHS/HPFS datasets to assess the associations of the HEI-2005 and the AHEI-2010 with major chronic diseases, including T2DM [12]. The main result indicates that the AHEI-2010 was more strongly associated with T2DM risk than the HEI-2005. Although both indices were significant, the association between HEI-2005 and T2DM risk was attenuated after adjustment for confounders [12]. In 2015, Jacobs and colleagues compared associations of the HEI-2010, AHEI-2010, DASH, and Alternate Mediterranean Diet Score with T2DM risk [17]. The study was conducted in the United States among men and women 45–75 years who participated in the Multiethnic Cohort Study. The main result indicates that the AHEI-2010 was associated with a 12% risk reduction of T2DM among white individuals. However, the HEI-2010 was not significantly associated with T2DM risk [17].

While there is growing evidence from prospective studies that high scores on the HEI or AHEI (corresponds to healthy dietary pattern) are inversely associated risk reduction of chronic disease, it remains unclear on whether the HEI-2010 or AHEI-2010 is preferable as a tool for dietary assessment in people with T2DM. Therefore, an improved understanding of the relationships between dietary pattern and health outcomes will help identify the appropriate tool to assess dietary quality for diabetes management and subsequently, decrease the risk of CHD and other diabetes-related complications.

In this study, the authors hypothesized that the AHEI-2010 is more strongly associated with T2DM than the HEI-2010 dietary pattern. To the authors’ knowledge, this was the first study that compared the HEI-2010 and AHEI-2010 scores and their associations with diabetes status in a representative sample of U.S. adults. Moreover, there is limited understanding of the differences of individuals’ dietary behavior at different stages of disease development. For that reason, the authors defined diabetes status into three categories: nondiabetes, prediabetes, and diabetes (T2DM). The authors were interested in looking at differences in dietary quality, and how they are associated with the stages of disease development. Furthermore, few studies have investigated the relationship between dietary pattern and physiological health markers. A better understanding of the biological basis of health markers (i.e., lipid profile) in relation to diet may better explain the differences in metabolism of individuals with and without chronic disease. In addition, this may provide an insight to develop more effective treatments for diabetes.

The main objectives of this study were three-fold: 1) To determine whether there were relationships between two measures of dietary quality, the HEI-2010 and AHEI-2010 and diabetes status (nondiabetes, prediabetes, T2DM); 2) To examine the relationships between the HEI-2010 and AHEI-2010 and health markers (including biomarkers); 3) To determine the strength of the relationships between the HEI-2010 and AHEI-2010 with diabetes status while controlling for health markers, lifestyle and demographic factors. All analyses were based on data from the 2007–2010 National Health and Nutrition Examination Survey (NHANES).

## Participants and methods

### Survey design

The NHANES is an ongoing cross-sectional survey that collects information on the health and nutritional status of the U.S. population [19]. The sample is representative of the civilian, non-institutionalized U.S. population because the participants are selected using complex, multistage, probability sampling design [20]. The CDC website provides complete details of the NHANES including study design, implementation, datasets, analytic considerations, and other documentations such as consent and operation manuals [19].

### Study sample

**Fig. 1:**
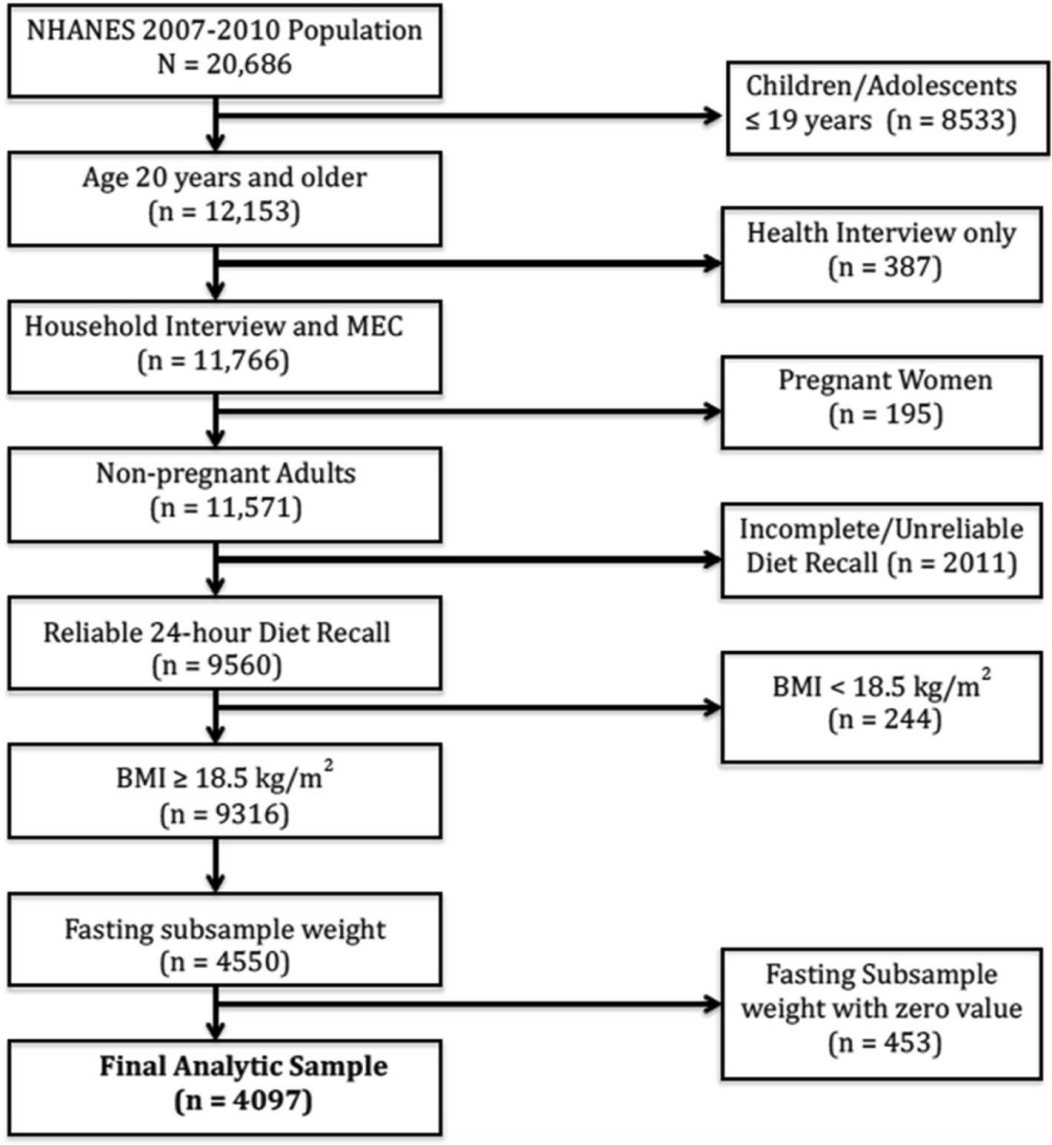
Study Sample using NHANES 2007–2010

The present study combined data from NHANES 2007–2008 and 2009–2010 to increase sample size. The study sample (*n* = 4097) was limited to adults age ≥ 20 years who participated in both the health interview and medical examination, self-reported as non-pregnant at the examination, had complete and reliable 24-h diet recalls, a Body Mass Index (BMI) ≥ 18.5 kg/m^2^, and fasting glucose measures during the morning examination session. (Fig. 1). NHANES is in compliance with federal law and follows stringent protocols and procedures that ensure confidentiality and protect participants’ identity [21]. A formal institutional review board approval was not required since this study was based on secondary analysis and did not contain any personal identifiers [22].

### Exposure and outcome variables

#### Estimation of dietary quality

This study utilized the Healthy Eating Index-2010 (HEI-2010) and the Alternate Eating Index-2010 (AHEI-2010) as the main exposure variables to assess dietary quality. The HEI-2010 and AHEI-2010 were calculated using the dietary intake data available in NHANES. Dietary intake information was obtained from two 24-h dietary recall interviews. The first recall was administered in-person at the Medical Examination Center (MEC) by trained interviewers using USDA’s Automated Multiple-Pass Method (AMPM). The second recall was administered via telephone interview, approximately 3 to 10 days after attending the MEC [23]. For reasons of methodology, interpretation, and comparability with other dietary surveys,^1^ this study used only data from the in-person recall (day 1) to calculate the HEI-2010 and AHEI-2010 scores. The use of the first day recall is recommended for most statistical analyses because the two dietary recalls cannot be considered as independent of one another. Combining them would underestimate the within-person variation and complicate the interpretation of the results. Furthermore, the use of two different methods to collect data (in-person for the first vs. telephone for the second) could affect participants’ responses and introduce bias. The varying length of time between recalls (3 to 10 days) may also introduce bias. Therefore, using the in-person (day 1) recall ensures consistency in dietary methodology and yields estimates that are most comparable with other dietary surveys. Additionally, this analysis was limited to dietary recall data reported to be complete and reliable by the National Center for Health Statistics staff [23].

##### Healthy Eating Index-2010 (HEI-2010)

The HEI-2010 was developed by the United States Department of Agriculture (USDA) Center for Nutrition Policy and Promotion (CNPP) as a tool to measure compliance with the 2010 Dietary Guidelines for Americans. In addition, the HEI-2010 is used to monitor the quality of American diets and examine relationships between diet and health-related outcomes [8]. The HEI-2010 is made up of 12 components: 9 components assess dietary adequacy (foods that people should consume more of) and 3 components assess moderation (foods that people should consume less of). For the moderation components, higher scores are associated with lower levels of consumption. The 9 adequacy components are: Total Fruit, Whole Fruit (forms other than juice), Total Vegetables, Greens and Beans (dark-green vegetables and beans and peas), Whole Grains, Dairy (all milk products and soy beverages), Total Protein Foods, Seafood and Plant Proteins, and Fatty Acids (ratio of poly- and monounsaturated fat to saturated fat). The 3 moderation components are: Refined Grains, Sodium, and Empty Calories (all calories from solid fats & added sugars plus calories from alcohol beyond a moderate level) [8]. Seven components were each scored on a 0 to 5 scale and the five other components are each scored on a 0 to 10 scale, with intermediate values scored proportionally. The component scores were summed to obtain total HEI-2010 scores. Higher scores indicate a higher quality diet. Scores above 80 indicate a “good” diet, while scores below 51 indicate a “poor” diet. An HEI score between 51 and 80 is considered as “needing dietary improvement” [8]. The Food Pattern Equivalents Database (FPED) was used to construct and calculate the HEI-2010 scores and the SAS code was downloaded from the USDA CNPP website [24].

##### Alternate Healthy Eating Index-2010 (AHEI-2010)

The AHEI-2010 was developed by researchers at the Harvard School of Public Health as an alternative measure of diet quality to identify future risk of diet-related chronic disease [7, 25, 26]. The AHEI was originally developed on the basis of the Food Frequency Questionnaire (FFQ). However, previous studies have used 24-h dietary recalls to compute the AHEI-2010 scores from NHANES [7, 25, 26]. In general, the continuous NHANES (since 1999) only collect 24-h recalls to assess dietary intake. For that reason, this study applied the methodology used Wang and colleagues (2014) to calculate the AHEI-2010 scores since the 2007–2010 NHANES only contain 24-h recalls for measuring dietary intake. The AHEI-2010 consists of 11 components: six components for which higher intakes are better [vegetables, fruit, whole grains, nuts and legumes, long chain omega-3 fatty acids (FA) that include docosahexaenoic acid and eicosapentaenoic acid, and Polyunsaturated Fatty Acids (PUFA)], one component for which moderate intake is better (alcohol), and four components that must be limited or avoided [sugar sweetened drinks and fruit juice, red and processed meat, trans fats, and sodium]. Each component was scored on a 0 to 10 point scale. The component scores were summed to obtain the total AHEI-2010 score, which can range from 0 (non-adherence) to 110 (perfect adherence). Higher scores represent healthier diets [10, 27]. However, this study constructed a modified AHEI-2010 score by excluding the trans fat component because trans fat is unavailable in the NHANES dietary files [24]. Therefore, the maximum total AHEI-2010 score was rescaled from 110 points to 100 points (excluding trans fat) similar to the approach used in a previous study [25, 26]. The NHANES individual foods file was used to estimate servings of food to construct the AHEI-2010 food groups. The USDA food-coding scheme was used as a reference to categorize each individual food (represented by food codes) into groups [28]. In addition, this study used the supplementary table provided by Wang and colleagues (2014) to identify the foods and beverages that correspond to each AHEI food component (i.e., sugar-sweetened beverages, nuts and legumes, red and/or processed meats) [7, 25, 26]. The NHANES total nutrients file was used to estimate the intake of nutrients (i.e., PUFA, long-chain omega-3 fats, sodium) as components of the AHEI. After creating the AHEI food and nutrient components, a SAS code was constructed to calculate the AHEI-2010 scores.

#### Diabetes status

T2DM status was the main outcome variable. The definition of T2DM was on the calculation of diagnosed and undiagnosed diabetes. Adults with diagnosed diabetes were defined as those who answered “yes” to the question: “Other than during pregnancy, have you ever been told by a doctor or other health professional that you have diabetes or sugar diabetes?” or who reported taking diabetes medication (i.e., Metformin) during the interview. Adults with undiagnosed diabetes were defined as individuals with a fasting plasma glucose (FPG) ≥ 126 mg/dL, or HbA1c ≥ 6.5% who did not report a previous diabetes diagnosis during the interview. The sum of individuals with diagnosed and undiagnosed diabetes was computed to obtain the total number of adults with T2DM. Individuals diagnosed with diabetes prior to age 30 and continuous users of insulin were excluded to minimize the number of respondents with type 1 diabetes (T1DM) [29, 30]. Adults with prediabetes were defined as those with FPG of 100–125 mg/dL, HbA1c 5.7–6.4%, or an answer of “yes” to the question “Have you ever been told by a doctor or other health professional that you have prediabetes?” or an answer of “borderline” to the question “Other than during pregnancy, have you ever been told by a doctor or other health professional that you have diabetes or sugar diabetes?” Participants who did not meet the definition for T2DM or pre-diabetes (FPG < 100 mg/dL and HbA1c < 5.7%) were categorized as nondiabetes [31].

#### Demographic and health characteristics

Demographic and health information were obtained from the household interview component of NHANES. Self-reported sociodemographic characteristics are explored as potential covariates. These included age, sex, ethnicity, education level, marital status, poverty-to-income ratio, adult food security category and health insurance coverage. Additional health and lifestyle factors that were considered to potentially influence diabetes included smoking status, physical activity, and self-reported health.

#### Health markers

Several health markers were evaluated in the analysis. These include Body Mass Index (BMI), Waist Circumference (WC), triglycerides (TG), total cholesterol, low-density lipoprotein cholesterol (LDL-C), high-density lipoprotein cholesterol (HDL-C), TG/HDL-C ratio, C-reactive protein (CRP), insulin, systolic and diastolic blood pressures, and presence of comorbidities (comorbidity score). Measurements of height, weight, WC, and blood pressure (systolic and diastolic) were obtained in the MEC according to the NHANES protocols [32]. BMI is calculated as body weight (in kilograms) divided by height (in meters) squared [33]. Data on physiological markers was obtained from laboratory testing of participants who attended the MEC; participants were randomly assigned to morning or afternoon sessions [34]. Venous blood was drawn to obtain lipid profile, CRP, insulin, and plasma glucose from fasting and non-fasting participants. Morning session participants had to fast for ≥8.5 h and were tested for LDL-C, triglycerides, insulin, and blood glucose. Afternoon session participants did not fast, and were tested only for total cholesterol and HDL-C. Therefore, not all participants have values for all laboratory tests [34]. This study limited the analysis to participants who provided fasting measures (i.e., serum LDL-C, insulin, and plasma glucose) by applying the nonzero fasting subsample weights [34, 35]. Blood pressure testing was also performed at the MEC. The majority of participants had at least three consecutive readings each for systolic and diastolic blood pressures. The fourth reading was taken in case the previous blood pressure measurement is interrupted or incomplete [36]. Therefore, this study only used the first three readings to calculate the average blood pressure (systolic and diastolic). The fourth reading was not included because it had a large number of missing values. Presence of comorbidities was evaluated using the medical conditions questionnaire. A comorbidity score (range: 0–15) was computed based on the sum of self-reported physician-diagnosed comorbidities that tend to co-occur with T2DM [31, 37, 38]. These comorbidities included overweight, high blood pressure, high cholesterol, heart attack, coronary heart disease, congestive heart failure, angina, stroke, thyroid problems, liver conditions, asthma, arthritis, chronic bronchitis, emphysema, and cancer/malignancy [37, 38].

### Statistical analysis

Data were analyzed using SAS 9.3 (SAS Institute Inc., Cary, NC) and STATA 14.1 (StataCorp, College Station, TX) to adjust the variances for the complex sample design of NHANES. To account for the complex multistage design, the 4-year fasting sample weight was used throughout the analysis in order to include participants who are already diagnosed with diabetes and taking insulin or oral medications. The fasting subsample weights (WTSAF2YR) for both cycles were used to construct a 4-year fasting weight as suggested in the NHANES analytic guidelines [35].

SAS (release 9.3) was the primary tool used in data preparation, cleaning, and analysis. The design-adjusted Rao-Scott chi-square test (PROC SURVEYFREQ) was used to compare participants’ sociodemographic characteristics by diabetes status. Linear regression (PROC SURVEYREG) was used to determine differences in total and sub-component HEI-2010 and AHEI-2010 scores across diabetes status (nondiabetes, prediabetes, diabetes) by calculating the Least-square means (LSMs). Least-square means (and the standard errors of the LSMs) were also calculated to determine differences in health markers across diabetes status and quartiles of total HEI-2010 and AHEI-2010 scores. For ease of presentation of the data, non-transformed LSMs were presented with *p*-values associated with transformed analyses. The covariates used for the LSMs were sex, ethnicity, age, poverty-to-income ratio, physical activity, and energy intake. Bonferroni correction for multiple comparisons (0.05/number of variables) was applied to obtain the effective p-values for the models. Binary logistic regression (PROC SURVEYLOGISTIC) was used to obtain predicted probabilities, which were then used to create a classification table showing the percentage of individuals correctly classified as to diabetes status based on each model specification. A cut-off of 0.5 was used to determine the predicted probability of T2DM. A predicted probability of 0.5 or greater indicated having T2DM and less than 0.5 indicated not having T2DM. Several models were developed using various predictors (including dietary quality, sociodemographics, health markers and lifestyle behaviors) with different specifications of diabetes status being categorized as a dichotomous outcome variable. Prediabetes is a risk factor for T2DM. Many people with prediabetes will eventually develop T2DM if interventions are not started early. Therefore, T2DM status was dichotomized by collapsing prediabetes with diabetes as the event and nondiabetes as the nonevent to determine whether or not the disease has occurred. All analyses had statistical significance set at *p* < 0.05.

STATA (release 14.1) software was used to perform multinomial logistic regression (svy: mlogit) to examine the association between dietary quality (using total HEI-2010 and AHEI-2010 scores) and diabetes status. T2DM status was used as a nominal outcome variable with three levels: nondiabetes, prediabetes, and diabetes. This categorization of T2DM status was specified in order to observe differences in the association of the HEI-2010 and AHEI-2010 scores among these subgroups. Separate models were specified for total HEI-2010 and AHEI-2010 scores after adjusting for sociodemographics, health markers, and lifestyle behaviors. The STATA command (mlogitgof) was executed after fitting the models to assess the goodness-of-fit for multinomial logit modeling [39].

#### Multivariate models

This study attempted to produce a model to explain the relationship between dietary quality (using total HEI-2010 and AHEI-2010 scores) and diabetes status including covariates on a theoretical basis rather than primarily relying on statistical significance. The covariates were selected based on previous studies of the association between dietary quality and T2DM. Covariates such as age, sex, ethnicity, smoking status, physical activity, WC, and energy intake appear consistently in regression models of previous studies [12, 14–16]. For that reason, predictors such as smoking status and physical activity were included in the models regardless of their statistical significance. Additional covariates such as self-reported health, poverty-to-income ratio, and presence of comorbidities were included since they had significant or marginally significant relationships with the probability of having diabetes (and prediabetes). Energy intake was included as a covariate for the AHEI-2010 because it is based on absolute amount of intake whereas the HEI-2010 already adjusts for energy intake using the density-based approach (amounts consumed per 1000 cal). In addition, several interaction terms were tested: these included WC × total HEI-2010 score, WC × total AHEI-2010 score, age × physical activity, physical activity × total HEI-2010 score, and physical activity × total AHEI-2010 score. These two-way interaction terms were not statistically significant and therefore were dropped from the models.

#### Classification of diabetes status using predicted probabilities

The next step was to specify models that represented different types of factors (including total and sub-component HEI-2010 and AHEI-2010 scores, sociodemographics, health markers, and health behaviors) and examine how well each model performed in correctly classifying participants by diabetes status. Sixteen binary logistic regression models were constructed to produce the predicted probabilities. For instance, as part of the model specification, this study sought to assess the predictive power of sociodemographics, health markers, and dietary quality individually and together (using HEI-210 and AHEI-2010 scores) by determining the percentage of the sample correctly classified as to diabetes status based on each of these factors. By identifying the contribution of these factors, this would provide a better guide to improve clinical practice in terms of prevention, treatment, and management of diabetes. Also, this study explored the predictive ability of the total and sub-component scores HEI-2010 and AHEI-2010 score in correctly classifying diabetes status. Only the sub-component scores for the HEI-2010 and AHEI-2010 that were significant in the bivariate analysis were included in the multivariate models (*p* < 0.05). For the HEI-2010, the sub-components included were total protein foods, refined grains, sodium, and empty calories. For the AHEI-2010, the sub-components included were sugar-sweetened beverages and fruit juice, red and/or processed meat, alcohol, and sodium. Knowing the predictive ability of these sub-components would provide more targeted dietary interventions for treating diabetes.

One of the major differences between the HEI-2010 and AHEI-2010 is the treatment of alcohol consumption. The HEI-2010 counts alcohol intake as part of empty calories (threshold exceeds moderate level more than 13 g/1000 kcal). However, the AHEI-2010 counts alcohol intake as a separate category where moderate drinking is a part of a healthful dietary pattern. The AHEI-2010 scoring methodology as reported by Wang et al. (2014) is non-linear and assigns higher scores to moderate alcohol drinkers than to nondrinkers [7]. Moderate alcohol drinkers (Male: 0.5–2.0 drinks/day; Female: 0.5–1.5 drinks/day) received the maximum score of 10 points, while nondrinkers received 2.5 points. This method of scoring severely penalizes nondrinkers. Therefore, this study explored two alternative approaches to scoring nondrinkers: 1) a deduction of 2.5 points from the maximum instead of 7.5; or 2) no penalty. Nondrinkers received the maximum score of 10 points, and scores declined as alcohol consumption increased. Each of these approaches was used to create modified AHEI-2010 scores. These modified alcohol scores were used in the multivariate analysis to determine whether there was a difference in their predictive ability compared to the original scoring of alcohol (score of 2.5 points for nondrinkers).

## Results

### Characteristics of the participants by diabetes status

Table 1 shows that the majority of sociodemographic characteristics were significantly associated with diabetes status (*p* < 0.01), with the exception of food security (*p* > 0.05). Individuals with prediabetes and diabetes were older, were more likely to be male, and reported themselves as currently married (*p* < 0.01). T2DM was strongly associated with educational level and other sociodemographics. The majority of diabetics reported having high school diploma (about 29.1%) and some college education (about 26.8%). Individuals with lower educational attainment were more likely to be diabetic than nondiabetic (i.e., about 25.1% vs. 12.9% with less than high school, respectively). Among ethnic minority groups, non-Hispanic blacks were more likely to have diabetes (about 15.7%). Nonsmokers were more likely to have diabetes than current and former smokers (about 49.5% vs. 13.5% vs. 36.9%, respectively). For self-reported health, individuals who rated their health as “good” (about 36.9%) or “fair” (about 32.3%) were more likely to have diabetes. Similarly, prediabetes was strongly associated with sociodemographics. Individuals with higher educational attainment were more likely to have prediabetes than diabetes (i.e., 28.2% vs. 18.9% with college graduate or above education, respectively). Among ethnic minority groups, non-Hispanic blacks were more likely to have diabetes (about 10.3%). Individuals with poverty-to-income ratio above 3.50 were more likely to have prediabetes (about 40.5%). Former smokers were more likely to have prediabetes than current smokers (about 27.9% vs. 19.2%, respectively). For self-reported health, individuals who rated their health as “very good” (about 31.9%) or “good” (about 37.1%) were more likely to have prediabetes. The majority of participants (including nondiabetics) in the sample did not engage in any physical activity. About 63.9% of adults with diabetes and 47.2% of adults with prediabetes were not physically active.

**Table 1:** Sociodemographic Characteristics of U.S. adults (Age ≥ 20 years) by Diabetes Status

Characteristic	(n)	^a^Diabetes Status						*p* trend
		Nondiabetes (*n* = 1436)		Prediabetes (*n* = 1905)		Diabetes (*n* = 715)		
	4056	*n*	%	*n*	%	*n*	%	
Age								**<.0001**
20–39		731	53.2	448	26.7	34	5.1	
40–59		459	34.9	704	43.3	219	38.2	
60–79		199	9.9	614	24.9	388	46.5	
80+		47	1.9	139	5.1	74	10.1	
Sex	4056							**<.0001**
Male		554	40.4	997	54.6	375	52.9	
Female		882	59.6	908	45.4	340	47.1	
Race/Ethnicity	4056							**0.0049**
Mexican American		255	7.8	338	8.0	136	8.2	
Non-Hispanic White		720	71.2	978	72.0	309	65.1	
Non-Hispanic Black		221	9.5	310	10.3	163	15.7	
Other		240	11.4	279	9.7	107	10.9	
Education Level	4050							**<.0001**
Less than high school		302	12.9	536	19.1	263	25.1	
High School diploma		311	20.2	459	25.1	188	29.1	
Some College education		428	30.7	501	27.6	172	26.8	
College Graduate or Above		395	36.1	405	28.2	90	18.9	
Marital Status	4055							**<.0001**
Current Married		832	62.0	1217	68.1	439	65.2	
Former Married		253	13.4	462	19.3	218	26.9	
Never Married		351	24.6	226	12.5	57	7.9	
Smoking Status	4056							**<.0001**
Nonsmoker		876	60.6	1008	52.9	353	49.5	
Current smoker		282	18.9	375	19.2	108	13.5	
Former smoker		278	20.4	522	27.9	254	36.9	
^b^Physical Activity	4056							**<.0001**
None		654	38.6	1020	47.2	489	63.9	
Insufficient		230	17.2	293	18.0	94	15.5	
Sufficient		552	44.1	592	34.8	132	20.6	
Poverty-to-Income Ratio	4056							**<.0001**
< 1.30		494	23.0	705	25.7	259	25.2	
1.30–3.49		498	32.9	649	33.8	300	43.6	
≥ 3.50		444	44.1	551	40.5	156	31.2	
Self-Reported Health	4055							**<.0001**
Excellent		280	23.3	224	14.1	32	6.0	
Very Good		457	37.4	535	31.9	97	15.6	
Good		481	29.2	719	37.1	242	36.9	
Fair		184	8.8	351	13.9	262	32.3	
Poor		34	1.4	75	3.0	82	9.2	
Covered by Health Insurance	4052							**0.0031**
Yes		1039	80.1	1455	82.1	604	88.1	
No		396	19.9	447	17.9	111	11.9	
Adult Food Security Category	4024							0.2948
Full		1045	82.3	1375	79.8	544	83.8	
Marginal		162	7.5	189	7.9	64	6.5	
Low		129	6.5	202	7.6	60	5.9	
Very Low		86	3.7	123	4.7	45	3.8	

Values are column percents *n* (%) for categorical variables by diabetes status. Statistical differences were assessed using design-based Rao-Scott F adjusted Χ^2^ statistic

Bolded values are significantly different *p* < 0.01

^a^Diabetes status was defined from self-report of participants in the diabetes questionnaire and from the laboratory biomarkers using the cut-offs based on the 2014 Standards of Medical Care from the American Diabetes Association (ADA) for diabetes diagnosis

^b^Physical Activity guidelines were defined for participants meeting (≥150 min/week of moderate-to-vigorous physical activity [MVPA]) or not meeting MVPA based on the 2008 Physical Activity Guidelines for Americans

### HEI-2010 total and components scores by diabetes status

Table 2 shows the HEI-2010 components and total scores by diabetes status. The total HEI-2010 score was highest for individuals with diabetes (mean score = 48.8 ± 0.6). However, these differences across diabetes status were not statistically significant (*p* > 0.05). Interestingly, there were significant differences in some of the component scores across diabetes status including total protein foods, refined grains, sodium, and empty calories (*p* < 0.01). For the adequacy components, adults with diabetes had the highest score (corresponding to highest intake) for total protein foods (mean score = 4.4 ± 0.06) compared to adults with prediabetes and nondiabetes. For the moderation components, adults with diabetes had the highest score (corresponding to lowest intake) for empty calories (mean score = 12.5 ± 0.3) compared to adults with prediabetes and nondiabetes. However, adults with prediabetes had the highest score for refined grains (mean score = 6.3 ± 0.1) and for sodium (mean score = 4.4 ± 0.07) compared to adults with prediabetes and nondiabetes (*p* < 0.01).

**Table 2:** HEI-2010 components and total scores of U.S. adults (Age ≥ 20 years) by Diabetes Status

Component	Criteria		Maximum Score Value	Diabetes Status			^a^p-value
	Minimum score	Maximum score		Nondiabetes (*n* = 1436)	Prediabetes (*n* = 1905)	Diabetes (*n* = 715)	
				LSM ± SE	LSM ± SE	LSM ± SE	
Adequacy							
Total fruit	0	≥0.8 cups/1000 kcal	5	2.1 ± 0.07	2.2 ± 0.08	2.2 ± 0.1	0.6460
Whole fruit	0	≥0.4 cups/1000 kcal	5	0.7 ± 0.04	0.8 ± 0.07	0.8 ± 0.08	0.0554
Total vegetables	0	≥1.1 cups/1000 kcal	5	2.9 ± 0.07	3.0 ± 0.04	3.2 ± 0.07	0.1362
Greens and beans	0	≥0.2 cups/1000 kcal	5	1.3 ± 0.06	1.2 ± 0.07	1.3 ± 0.1	0.5413
Whole grains	0	≥1.5 oz./1000	10	2.5 ± 0.1	2.2 ± 0.1	2.7 ± 0.1	0.0693
Dairy	0	≥1.3 cups/1000 kcal	10	5.4 ± 0.1	5.1 ± 0.09	5.1 ± 0.2	0.1506
Fatty acids	(PUFAs + MUFAs)/ SFAs ≤1.2	(PUFAs + MUFAs)/ SFAs ≥2.5	10	4.9 ± 0.1	4.9 ± 0.1	5.1 ± 0.2	0.6415
Total protein foods	0	≥2.5 oz./1000 kcal	5	4.2 ± 0.04	4.2 ± 0.04	4.4 ± 0.06	**0.0008**
Seafood and Plant proteins	0	≥0.8 oz./1000 kcal	5	2.2 ± 0.1	2.0 ± 0.08	1.9 ± 0.1	0.1646
Moderation							
Refined grains	≥ 4.3 oz./1000 kcal	≤ 1.8 oz./1000 kcal	10	5.9 ± 0.1	6.3 ± 0.1	5.9 ± 0.2	**0.0122**
Sodium	≥ 2.0 g/1000 kcal	≤ 1.1 g/1000 kcal	10	4.2 ± 0.08	4.4 ± 0.07	3.5 ± 0.2	**0.0006**
^b^Empty calories	≥ 50% of energy	≤ 19% of energy	20	11.1 ± 0.3	10.4 ± 0.3	12.5 ± 0.3	**0.0002**
Total HEI-2010 score			100	47.5 ± 0.6	46.8 ± 0.7	48.8 ± 0.6	0.1110

Values are least square means ± standard error of the mean (SE)

All scoring criteria were calculated per 1000 kcal/d, except empty calories, which are calculated as % total energy. For adequacy components, higher intake of food/nutrient groups result in higher scores. For moderation components, lower intake of food/nutrient groups result in higher scores

*Abbreviations*: *HEI-2010* Healthy Eating Index 2010, *LSM* least square means, *MUFA* monounsaturated fatty acid, *PUFA* polyunsaturated fatty acid, *SFA* saturated fatty acid

^a^Bonferroni correction (< 0.05/12 HEI-2010 components), *P* < 0.004

^b^Empty Calories from solid fats, alcohol, and added sugars; threshold for counting alcohol is more than 13 g/1000 kcal

Bold data indicate statistically different *p* < 0.05

### AHEI-2010 total and components scores by diabetes status

Table 3 shows the AHEI-2010 components and total scores by diabetes status. The total AHEI-2010 score was highest for adults with diabetes (mean score = 39.1 ± 0.7). However, these differences across diabetes status were not statistically significant (*p* > 0.05). Interestingly, there were significant differences in some of the component scores across diabetes status including sugar-sweetened beverages and fruit juice, red and/or processed meats, alcohol, and sodium (*p* < 0.01). Adults with diabetes had the highest score (corresponding to lowest intake) for sugar-sweetened beverages and fruit juice (mean score = 3.3 ± 0.2) and sodium (mean score = 6.1 ± 0.2) compared to adults with prediabetes and nondiabetes. In addition, adults with diabetes had the lowest score (corresponding to highest intake) for red and/or processed meat (mean score = 5.9 ± 0.2). Adults with diabetes had the lowest score (corresponding to lowest intake) for alcohol (mean score = 2.8 ± 0.07) compared to prediabetics and nondiabetics.

**Table 3:** AHEI-2010 components and total scores of U.S. adults (Age ≥ 20 years) by Diabetes Status

Component	Criteria		Maximum Score value	Diabetes Status			^a^p-value
	Minimum score	Maximum score		Nondiabetes (*n* = 1436)	Prediabetes (*n* = 1905)	Diabetes (*n* = 715)	
				LSM ± SE	LSM ± SE	LSM ± SE	
Whole fruit	0	≥4 servings/d	10	2.8 ± 0.1	3.0 ± 0.1	2.9 ± 0.2	0.6109
Total vegetables	0	≥2.5 cups/d	10	2.6 ± 0.1	2.8 ± 0.1	2.9 ± 0.2	0.2947
Whole grains	0	Women: 75 g/d Men: 90 g/d	10	2.9 ± 0.1	2.9 ± 0.2	3.3 ± 0.2	0.3386
Sugar-sweetened Beverages and fruit juice	≥8 oz./d	0	10	2.4 ± 0.2	2.5 ± 0.1	3.3 ± 0.2	**0.0011**
Nuts and legumes	0	≥1 oz./d	10	2.5 ± 0.2	2.4 ± 0.1	2.4 ± 0.2	0.8478
Red and/or Processed Meats	≥1.5 servings/d	0	10	6.5 ± 0.2	6.0 ± 0.1	5.9 ± 0.2	**0.0 318**
Long-chain (ω-3) fats (EPA + DHA)	0	250 mg/d	10	2.6 ± 0.1	2.8 ± 0.1	2.3 ± 0.2	0.0937
PUFAs	≤2% of energy	≥10% of energy	10	6.9 ± 0.05	6.9 ± 0.05	7.2 ± 0.1	0.0772
^b^Alcohol	Men: ≥3.5 drinks/d Women: ≥2.5 drinks/d	Men: 0.5–2.0 drinks/d Women: 0.5–1.5 drinks/d	10	3.4 ± 0.09	3.3 ± 0.08	2.8 ± 0.07	**<.0001**
^c^Sodium	Highest Decile	Lowest Decile	10	5.2 ± 0.1	5.6 ± 0.1	6.1 ± 0.2	**0.0017**
Total AHEI-2010 score			100	37.9 ± 0.7	38.3 ± 0.5	39.1 ± 0.7	0.4299

Values are least square means ± standard error of the mean (SE)

All scoring criteria were calculated based on actual intake of participants rather than absolute standards. Trans fat component was omitted from the AHEI-2010 scoring because it is unavailable in the NHANES dietary files. For sugar-sweetened beverages and fruit juices, red and/or processed meat, and sodium, a higher score corresponds to lower intake

*Abbreviations*: *AHEI-2010* Alternate Healthy Eating Index-2010, *LSM* least square means, *DHA* docosahexaenoic acid, *EPA* eicosapentaenoic acid, *PUFA* polyunsaturated fatty acid

^a^Bonferroni correction (< 0.05/11 AHEI-2010 components), *P* < 0.005

^b^Alcoholic drinkers were assigned the highest score to moderate, and lowest score to heavy consumers. Nondrinkers received a score of 2.5

^c^Consumption was based on the actual intake distribution of participants in the sample. The fasting subsample weight was used to obtain representative percentiles for sodium intake in the sample

Bold data indicate statistically different *p* < 0.05

### Health markers and HEI-2010 and AHEI-2010 scores

There was a significant linear trend between the quartiles of total HEI-2010 and AHEI-2010 scores and health markers (Tables 4 and 5). With increasing HEI-2010 and AHEI-2010 scores, there was a significant linear decrease in BMI, WC, triglycerides, TG/HDL cholesterol ratio, and presence of comorbidities (*p* < 0.01). Also, there was a significant linear increase in HDL cholesterol across quartiles of HEI-2010 and AHEI-2010 scores (*p* < 0.01) (Tables 4 and 5). In addition, the mean concentration of LDL cholesterol and mean systolic blood pressure significantly decreased with increased total AHEI-2010 score (*p* = 0.0406 and *p* = 0.0109, respectively) (Table 5**)**.

**Table 4:** Association between total HEI-2010 score and Health Markers in U.S. adults (Age ≥ 20, *N* = 4097)

Health Markers	(n)	HEI-2010 Quartiles				*p* trend
		Quartile 1 (*n* = 982)	Quartile 2 (*n* = 1066)	Quartile 3 (*n* = 1014)	Quartile 4 (*n* = 1035)	
		LSM ± SE	LSM ± SE	LSM ± SE	LSM ± SE	
^a^BMI (kg/m^2^)	4097	29.5 ± 0.3	29.5 ± 0.3	28.9 ± 0.2	28.3 ± 0.3	0.0041*
^a^WC (cm)	4019	101.1 ± 0.5	100.5 ± 0.6	99.3 ± 0.5	97.9 ± 0.6	0.0014*
^a^Total cholesterol (mg/dl)	4070	201.0 ± 2.1	196.1 ± 1.6	194.7 ± 1.2	196.6 ± 1.5	0.0875*
^b^HDL cholesterol (mg/dl)	4070	51.7 ± 0.7	53.8 ± 0.6	52.4 ± 0.6	54.9 ± 0.5	0.0014*
^a^LDL cholesterol (mg/dl)	3990	120.7 ± 1.7	116.9 ± 1.5	115.5 ± 0.9	115.4 ± 1.2	0.0987
^a^TG (mg/dl)	4066	150.7 ± 4.9	130.2 ± 3.5	134.8 ± 4.3	129.5 ± 3.1	0.0001*
^a^TG / HDL cholesterol Ratio	4066	3.6 ± 0.2	2.9 ± 0.1	3.1 ± 0.2	2.9 ± 0.1	0.0002*
^c^CRP (mg/dl)	4086	0.5 ± 0.03	0.4 ± 0.02	0.4 ± 0.02	0.4 ± 0.03	0.1221*
^c^Insulin (uU/mL)	3958	13.8 ± 0.5	13.5 ± 0.4	13.5 ± 0.4	12.6 ± 0.3	0.2315*
^c^Mean SBP (mm Hg)	3928	122.6 ± 0.5	122.2 ± 0.5	122.4 ± 0.6	120.9 ± 0.6	0.1905
^c^Mean DBP (mm Hg)	3928	68.2 ± 0.5	69.0 ± 0.5	68.3 ± 0.5	69.0 ± 0.6	0.4356
^a^Comorbidity Score	4097	2.2 ± 0.1	2.1 ± 0.05	2.0 ± 0.06	1.9 ± 0.07	0.0167

Values are least square means ± standard error of the mean (SE). Bonferroni correction (< 0.05/12 health markers), *P* < 0.004

Adjusted for age, sex, ethnicity, smoking status, PIR, physical activity, and energy intake

Highest total HEI-2010 score was compared to the lowest total HEI-2010 (quartile 4 vs. quartile 1) to represent dietary quality. Mean blood pressure (systolic and diastolic) was calculated based on the average of the first three systolic BP readings of the participants while being examined at the MEC. The fourth reading was not included because of missing values. Co-morbidity score was calculated as the sum of self-reported presence of physician-diagnosed comorbidities that tend to co-occur with type 2 diabetes

*Abbreviations*: *HEI-2010* Healthy Eating Index 2010, *LSM* Least-Square Means, *BMI* Body Mass Index, *WC* Waist Circumference, *TC* Total Cholesterol, *HDL-C* High density Lipoprotein Cholesterol, *LDL-C* Low density Lipoprotein Cholesterol, *TG* Triglycerides, *CRP* C-reactive protein

^b^Adjusted for age, sex, ethnicity, smoking status, Poverty-to-Income Ratio, physical activity, energy intake, and BMI

^c^Adjusted for age, sex, ethnicity, smoking status, Poverty-to-Income Ratio, physical activity, energy intake, BMI and WC

*Nontransformed LSMs were presented with *p*-values associated with the variables after log-transformation for normality. Health markers that were log-transformed include BMI, WC, TC, HDL-C, TG, CRP, and Insulin. Bolded values are significantly different *p* < 0.01

**Table 5:** Association between total AHEI-2010 score and Health Markers in U.S. adults (Age ≥ 20, N = 4097)

Health Markers	(n)	AHEI-2010 Quartiles				*p* trend
		Quartile 1 (*n* = 990)	Quartile 2 (*n* = 1003)	Quartile 3 (*n* = 1062)	Quartile 4 (*n* = 1042)	
		LSM ± SE	LSM ± SE	LSM ± SE	LSM ± SE	
^a^BMI (kg/m^2^)	4097	29.9 ± 0.2	29.2 ± 0.3	29.1 ± 0.2	27.9 ± 0.3	0.0011*
^a^WC (cm)	4019	101.8 ± 0.5	100.7 ± 0.6	99.4 ± 0.6	96.9 ± 0.7	0.0003*
^a^Total cholesterol (mg/dl)	4070	199.3 ± 1.8	197.1 ± 1.6	197.8 ± 1.6	194.4 ± 1.4	0.1790*
^b^HDL cholesterol (mg/dl)	4070	51.6 ± 0.7	52.1 ± 0.5	53.8 ± 0.6	55.5 ± 0.6	<. 0001*
^a^LDL cholesterol (mg/dl)	3990	119.7 ± 1.6	118.6 ± 1.3	116.8 ± 1.3	113.6 ± 1.3	0.0406
^a^TG (mg/dl)	4066	148.0 ± 5.1	134.4 ± 2.7	136.1 ± 3.1	125.9 ± 3.8	<. 0001*
^a^TG / HDL cholesterol Ratio	4066	3.5 ± 0.2	2.9 ± 0.07	3.1 ± 0.1	2.9 ± 0.2	<. 0001*
^c^CRP (mg/dl)	4086	0.4 ± 0.03	0.4 ± 0.03	0.4 ± 0.02	0.4 ± 0.02	0.0054*
^c^Insulin (uU/mL)	3958	13.9 ± 0.3	13.4 ± 0.3	13.2 ± 0.4	12.7 ± 0.2	0.0940*
^c^Mean SBP (mm Hg)	3928	122.7 ± 0.5	122.8 ± 0.6	121.8 ± 0.6	120.6 ± 0.5	0.0109
^c^Mean DBP (mm Hg)	3928	68.1 ± 0.6	68.9 ± 0.5	68.3 ± 0.6	69.2 ± 0.5	0.4184
^a^Comorbidity Score	4097	2.3 ± 0.09	2.1 ± 0.09	2.0 ± 0.09	1.9 ± 0.09	**<.0001**

Values are least square means ± standard error of the mean (SE). Bonferroni correction (< 0.05/11 health markers), *P* < 0.005

Highest total AHEI-2010 score was compared to the lowest total AHEI-2010 (quartile 4 vs. quartile 1) to represent dietary quality. Mean blood pressure (systolic and diastolic) was calculated based on the average of the first three systolic BP readings of the participants while being examined at the MEC. The fourth reading was not included because of missing values. Co-morbidity score was calculated as the sum of self-reported presence of physician-diagnosed comorbidities that tend to co-occur with type 2 diabetes

*Abbreviations*: *AHEI-2010* Alternate Healthy Eating Index 2010, *LSM* Least-Square Means, *BMI* Body Mass Index, *WC* Waist Circumference, *TC* Total Cholesterol, *HDL-C* High density Lipoprotein Cholesterol, *LDL-C* Low density Lipoprotein Cholesterol, *TG* Triglycerides, *CRP* C-reactive protein

^a^Adjusted for age, sex, ethnicity, smoking status, PIR, physical activity, and energy intake

^b^Adjusted for age, sex, ethnicity, smoking status, Poverty-to-Income Ratio, physical activity, energy intake, and BMI

^c^Adjusted for age, sex, ethnicity, smoking status, Poverty-to-Income Ratio, physical activity, energy intake, BMI and WC

*Nontransformed LSMs were presented with p-values associated with the variables after log-transformation for normality. Health markers that were log-transformed include BMI, WC, TC, HDL-C, TG, CRP, and Insulin. *P*-value is not significant for CRP without log-transformation. Bolded values are significantly different *p* < 0.01

### Health markers and diabetes status

Table 6 shows the covariate-adjusted health markers of participants by diabetes status. The majority of health markers were significantly associated with diabetes status after adjusting for age, sex, ethnicity, smoking status, poverty-to-income ratio, physical activity level, and energy intake (*p* < 0.001). Compared with prediabetes and nondiabetes, adults with diabetes had significantly higher mean BMI, WC, triglycerides, TG/HDL cholesterol ratio, insulin, mean systolic blood pressure, and total co-morbidities (*p* < 0.001). However, adults with prediabetes had significantly higher mean total cholesterol and LDL cholesterol (*p* < 0.001) compared to adults with nondiabetes (and diabetes). There were no significant differences in C-reactive protein across diabetes status groups (*P* > 0.05).

**Table 6:** Association between Diabetes Status and Health Markers in U.S. adults (Age ≥ 20 years)

Health Markers	(n)	Diabetes Status			^a^p-value
		Nondiabetes (n = 1436)	Prediabetes (*n* = 1905)	Diabetes (*n* = 715)	
		LSM ± SE	LSM ± SE	LSM ± SE	
^a^BMI (kg/m^2^)	4056	26.7 ± 0.2	29.6 ± 0.2	32.8 ± 0.4	<.0001*
^a^WC (cm)	3978	93.9 ± 0.6	100.9 ± 0.4	108.8 ± 0.9	<.0001*
^a^Total cholesterol (mg/dl)	4029	197.6 ± 1.5	201.9 ± 1.0	181.1 ± 2.5	<.0001*
^b^HDL cholesterol (mg/dl)	4029	55.5 ± 0.5	52.9 ± 0.5	49.3 ± 0.6	<.0001*
^a^LDL cholesterol (mg/dl)	3952	117.3 ± 1.0	122.1 ± 0.9	101.3 ± 1.8	**<.0001**
^a^TG (mg/dl)	4025	116.9 ± 2.8	139.2 ± 2.7	168.8 ± 8.3	<.0001*
^a^TG / HDL cholesterol Ratio	4025	2.3 ± 0.08	3.2 ± 0.1	4.5 ± 0.3	**<.0001**
^b^CRP (mg/dl)	4045	0.4 ± 0.01	0.4 ± 0.03	0.4 ± 0.02	0.2205
^c^Insulin (uU/mL)	3918	11.1 ± 0.3	14.1 ± 0.3	15.9 ± 0.7	<.0001*
^c^Mean SBP (mm Hg)	3888	120.0 ± 0.4	122.4 ± 0.5	124.9 ± 1.1	**<.0001**
^c^Mean DBP (mm Hg)	3888	67.8 ± 0.7	69.4 ± 0.4	68.0 ± 0.7	0.0155
^a^Comorbidity Score	4056	1.7 ± 0.09	2.0 ± 0.09	3.0 ± 0.1	**<.0001**

Values are least square means ± standard error of the mean (SE). Bonferroni correction (< 0.05/12 health markers), *P* < 0.004

Mean blood pressure (systolic and diastolic) was calculated based on the average of the first three systolic BP readings of the participants while being examined at the MEC. The fourth reading was not included because of missing values. Co-morbidity score was calculated as the sum of self-reported presence of physician-diagnosed comorbidities that tend to co-occur with type 2 diabetes

*Abbreviations*: *LSM* Least-Square Means, *BMI* Body Mass Index, *WC* Waist Circumference, *TC* Total Cholesterol, *HDL-C* High density Lipoprotein Cholesterol, *LDL-C* Low density Lipoprotein Cholesterol, *TG* Triglycerides, *CRP* C-reactive protein

^a^Adjusted for age, sex, ethnicity, smoking status, Poverty-to-Income Ratio, physical activity, and energy intake

^b^Adjusted for age, sex, ethnicity, smoking status, Poverty-to-Income Ratio, physical activity, energy intake, and BMI

^c^Adjusted for age, sex, ethnicity, smoking status, Poverty-to-Income Ratio, physical activity, energy intake, BMI and WC

*Nontransformed LSMs were presented with p-values associated with the variables after log-transformation for normality. Health markers that were log-transformed include BMI, WC, TC, HDL-C, TG, CRP, and Insulin. Bolded values are significantly different *p* < 0.01

### Association between HEI-2010 and AHEI-2010 scores and diabetes status

Results from the multinomial logistic models show that the odds ratios for total HEI-2010 and AHEI-2010 scores is equal to 1, which means that they have no predictive value for diabetes status. Contrary to the study hypothesis, the models suggest that the AHEI-2010 does not seem to perform better than the HEI-2010 in terms of its association with T2DM (Additional file 1). Table 7 shows the classification of diabetes status using predicted probabilities. Results show that the model with only sociodemographic characteristics (Model 1) classified the largest percentage of the sample correctly with respect to diabetes status (about 65.1% correct classification) and lower percentages of false positive (about 24.2%) and false negative (about 10.7%). The model with only the health markers (Model 2) also classified the majority of respondents correctly (about 63.3% correct classification). However, the models with only the HEI-2010 and AHEI-2010 scores (total and sub-components) only classified about 52% of the sample correctly (Model 5, 6, 7, 11, 12, and 13). This means that dietary quality alone (using both total and sub-component HEI-2010 and AHEI-2010 scores) is not a good predictor of diabetes status. Interestingly, the model with dietary quality and other health behaviors (i.e., smoking status, and physical activity) increased predictive ability slightly, with about 54% of the sample correctly classified diabetes (Model 3 and 4). The AHEI-2010 score did not perform any better than the HEI-2010 score in terms of predicting or explaining diabetes.

**Table 7:** Classification of Diabetes Status among U.S. adults (Age ≥ 20 years) using Predicted Probabilities

Models	% correctly classified	% false positive	% false negative
1	65.1	24.2	10.7
2	63.3	7.0	29.6
3	53.9	42.1	4.0
4	54.6	40.5	4.9
5	51.9	48.1	0
6	51.9	47.9	0.1
7	51.9	47.9	0.1
8	63.7	6.2	30.2
9	63.6	6.1	30.3
10	63.7	6.1	30.3
^a^11	51.9	47.9	0.2
^b^12	52.7	44.8	2.5
^c^13	52.9	44.5	2.6
14	63.5	6.3	30.2
15	63.5	6.2	30.3
16	63.6	6.1	30.3

Values are percentages

Diabetes status was recoded as a binary outcome. False negative is when the predicted probability of having diabetes/prediabetes wrongly classifies diabetes/prediabetes as the nonevent. False positive is when the predicted probability of having diabetes/prediabetes wrongly classifies diabetes/prediabetes as the event

Model 1 = Sociodemographics (age, sex, ethnicity, Poverty-to-Income Ratio, self-reported health)

Model 2 = Health Markers (Comorbidity Score, mean systolic BP, WC, LDL-C, HDL-C)

Model 3 = Health Behaviors (total HEI-2010 score + physical activity + smoking status)

Model 4 = Health Behaviors (total AHEI-2010 score + physical activity + smoking status)

Model 5 = Total HEI-2010 score

Model 6 = Total AHEI-2010 score

Model 7 = Modified Total AHEI-2010 score (no alcohol penalty)

Model 8 = Model 1 + Model 2 + Total HEI-2010 score

Model 9 = Model 1 + Model 2 + Total AHEI-2010 score

Model 10 = Model 1 + Model 2 + Modified Total AHEI-2010 score (no alcohol penalty)

Model 11 = HEI-2010 components

Model 12 = AHEI-2010 components

Model 13 = AHEI-2010 components (no alcohol penalty)

Model 14 = Model 1 + Model 2 + HEI-2010 components

Model 15 = Model 1 + Model 2 + AHEI-2010 components

Model 16 = Model 1 + Model 2 + AHEI-2010 components (no alcohol penalty)

*Abbreviations*: *BP* Blood Pressure, *WC* Waist Circumference, *LDL-C* Low density Lipoprotein Cholesterol, *HDL-C* High density Lipoprotein Cholesterol, *HEI-2010* Healthy Eating Index 2010, *AHEI-2010* Alternate Healthy Eating Index 2010

^a^Selected HEI-2010 components that are significantly associated with diabetes status (total protein foods, refined grains, sodium, empty calories)

^b^Selected AHEI-2010 components that are significantly associated with diabetes status (sugar-sweetened beverages and fruit juice, red/processed meat, alcohol, sodium)

^c^Replaced the original scoring of alcohol as 2.5 for nondrinkers with a modified scoring of alcohol as zero for nondrinkers (no alcohol penalty)

## Discussion

The results of present study were not consistent with the results of earlier cross-sectional studies that compared the HEI and AHEI scores in relation to T2DM. The main result of this study was that the AHEI-2010 did not to perform better than the HEI-2010 in terms of its relationship with diabetes status. This was in contrast with the results of a cross-sectional study by Huffman and colleagues that examined the relationship between the HEI-2005 and the AHEI-2005 scores and 10-year predicted CHD risk in Cuban Americans with and without T2DM [40]. The authors performed hierarchical linear regression models and used diabetes status as one of the covariates to predict CHD risk. They found that for every unit increase in the AHEI-2005 score, there was a 0.24-point reduction in the 10-year CHD risk score among participants with T2DM. However, they did not find a significant association between HEI-2005 score and CHD risk among participants without T2DM [40]. Another similar study by Huffman and colleagues assessed the relationships of the HEI-2005 and AHEI-2005 among Haitian Americans (HA) and African Americans (AA) with and without T2DM [41]. They found that the HEI-2005 score was significantly higher among individuals with diabetes (T2DM) compared to nondiabetes after controlling for age, gender, ethnicity, and education. However, the difference in AHEI-2005 scores among individuals with diabetes and nondiabetes was not significant [41].

There are several possible ways to interpret the apparent inconsistencies between the results of these earlier cross-sectional studies and the present study. First, this study used total the HEI and AHEI scores based on the 2010 dietary guidelines and evidence-based recommendations rather than the 2005 guidelines. Second, this study and the earlier studies focused on different outcomes. This study used logistic regression to examine diabetes status as the dependent variable and the HEI-2010 and AHEI-2010 scores as the independent variables. Third, this study used differences in the HEI-2010 and AHEI-2010 by diabetes status and did not further assess the health risks of individuals with diabetes (T2DM). The AHEI-2010 is based on current knowledge of dietary factors that mainly contribute to CVD (i.e., myocardial infarction, angina, stroke, transient ischemic attack, and revascularization) [11, 12]. T2DM is associated with increased risk of CVD and is an independent risk factor for CHD [4, 40]. This may indicate that the AHEI-2010 would be more applicable among diabetic individuals with pre-existing CVD conditions.

Some prospective studies have found significant inverse associations between the HEI-2010 and AHEI-2010 scores and risk of T2DM [11, 14–17]. The association was found to be stronger for the AHEI-2010 than for the HEI-2010 in relation to T2DM. These studies found that greater adherence to the AHEI-2010 dietary pattern was associated with 23–36% risk reduction in T2DM [14, 17]. However, the present study did not confirm earlier findings of significant association of the HEI-2010 nor the AHEI-2010 in relation to T2DM. A possible reason could be differences in how diet was assessed (i.e., 24-h recall vs. FFQ) to calculate the HEI-2010 and AHEI-2010 scores. This study used a single 24-h dietary recall to calculate the HEI-2010 and AHEI-2010 scores. Therefore, measuring dietary quality based on one or even two days of intake may not serve as a good predictor of chronic disease (i.e., diabetes) that takes years to develop. However, assessing dietary quality based on habitual or usual intake may serve as a better predictor. It might be possible to find a significant relationship with diabetes status if the HEI-2010 and AHEI-2010 scores were calculated based on the FFQ since it is designed to evaluate usual dietary intake. NHANES uses the 24-h recall rather than the FFQ to capture food intake. This study attempted to replicate the methods from previous studies that used the 24-h dietary recalls to compute the HEI-2010 and AHEI-2010 scores from NHANES [7, 25, 26, 42, 43]. NHANES is currently considered to be the best source of valid and reliable data on dietary intake.

Additionally, there is inconsistency in modeling decisions and specification when examining the association between diet and disease (i.e., T2DM). In epidemiology, some studies attempt to specify models that are parsimonious while other studies control for a large number of variables. When variables are intercorrelated (as socioeconomic and demographic characteristics often are), this can lead to multicollinearity if multiple variables are entered into the model. In the present study, the AHEI-2010 score did not provide any improvement over the HEI-2010 in terms of predicting or explaining T2DM (and prediabetes) after adjusting for potential covariates. A possible reason is the interrelationships among the covariates that are included in the multivariate models (i.e., health markers and lifestyle characteristics). For instance, smoking status was significantly associated with physical activity, body size (as measured by WC), and presence of comorbidities (as measured by total comorbidity score). Also, dietary quality (using the total HEI-2010 and AHEI-2010 scores) seemed to be related to the other predictors, which makes it difficult to construct a definitive model that determines the effect of dietary quality alone in relation to diabetes status. The predicted probabilities suggest that the models specified for only the HEI-2010 and AHEI-2010 scores (total and sub-components) classified the least percentage of the sample correctly (about 52% correct classification) with respect to diabetes status compared to the other factors (i.e., sociodemographics, health markers). Classification of diabetes status did not improve when adding more variables to the models possibly because of the interrelationships among the variables. In addition, the percentage of false negative (i.e., when results indicate a person does not have the disease but actually does have the disease) increases when adding more variables to the models. Therefore, the true predictive value of dietary quality (using HEI-2010 and AHEI-2010) is not observed in relation to diabetes status. Diet is a complex exposure variable. There are numerous factors that influence diet, which in turn can have an impact on disease development (i.e., T2DM). This calls for more consistency in model specification, and maybe alternative approaches, to examine the relationship between diet and disease.

Both the HEI-2010 and AHEI-2010 scores indicated that U.S. adults need improvement in dietary pattern (mean total HEI-2010 score = 47.3 ± 0.4; mean total AHEI-2010 score = 38.2 ± 0.4). Adults with diabetes appeared to have more healthful dietary patterns (as shown by higher total scores) compared to adults with prediabetes and nondiabetes. It is likely that participants with diabetes are receiving more regular health care than other groups. Participants with diabetes (diagnosed) with regular doctor visits are more closely followed and receive nutrition counseling and are taught self-management skills to improve their health.

The HEI-2010 and AHEI-2010 individual food and nutrient component scores are clinically important because they can provide more insight about dietary quality, which would allow more flexibility to tailor dietary intervention among individuals with diabetes. This study found statistically significant differences in the sub-component HEI-2010 and AHEI-2010 scores across diabetes status (Tables 2 and 3). Some of the food and nutrient groups in the HEI-2010 and AHEI-2010 were aligned with one another in terms of protein and carbohydrate intake. For example, adults with diabetes had the highest intake of total protein foods (corresponding to highest score) in the HEI-2010 (Table 2), which was consistent with their having the highest intake of red and/or processed meat (corresponding to lowest score) in the AHEI-2010 (Table 3). Similarly, adults with diabetes had the lowest intake of empty calories (corresponding to highest score) in the HEI-2010 (Table 2), which was consistent with their having the lowest intakes of alcohol (corresponding to lowest score) and sugar-sweetened beverages and fruit juice (corresponding to highest score) in the AHEI-2010 (Table 3). In terms of clinical relevance, it seems that adults with diabetes are consuming food groups that are higher in protein and lower in carbohydrates and fats than other groups.

A possible explanation is that individuals with T2DM receive regular care and are counseled to avoid consuming excessive carbohydrates. As part of diabetes self-management, they are being taught to monitor their carbohydrate intake through carbohydrate counting, or “carb counting,” which is a meal planning technique for managing blood glucose levels in balance with medication or insulin intake and physical activity [31]. In addition, individuals with T2DM are more likely to consume a low-fat diet as recommended by the American Diabetes Association (ADA) and American Heart Association [31, 44]. As a result, the decrease in carbohydrate or fat intakes involves a compensatory increase in protein intake.

High protein diets such as the Atkins, South Beach, and Paleo diets are recommended weight reduction methods because protein reduces hunger, improves satiety, and increases thermogenesis [45]. Also, when combined with a reduction in calories, high protein diets enhance weight reduction while maintaining lean muscle mass [45]. Several studies have shown the benefits of a modest increase in dietary protein intake among individuals with diabetes with normal renal function [46–48]. Higher dietary protein consumption has a favorable effect on CVD risk factors among individuals with T2DM. It is associated with reduction in HbA1c, total serum cholesterol, LDL cholesterol, triglycerides, blood pressure, and C-reactive protein. It is also associated with an increase in HDL cholesterol [46–48]. In clinical practice, T2DM patients affected by overweight and obesity and have normal renal function are often advised to increase absolute protein intake to 1.5–2 g/kg of body weight (or 20–30% of total caloric intake) during weight reduction [45]. However, the ADA recommends that dietary protein not exceed between 1 and 1.5 g/kg of body weight (or 15–20% of total caloric intake) [31]. Despite the potential health benefits, high protein intake may have adverse long-term effects on renal function in individuals with diabetes (as well as in healthy individuals).

The present study demonstrated that adults with diabetes consumed less sugar and alcohol compared to adults with prediabetes and nondiabetes. The HEI-2010 and AHEI-2010 differ in how they assess the intakes of sugar and alcohol in the diet. In HEI-2010, sugar and alcohol intake were summed and counted as empty calories. Empty calories are composed of all calories from solid fats, added sugars, and alcohol intake beyond a moderate level (more than 13 g /1000 cal). In the AHEI-2010, sugar and alcohol are considered to be separate categories. Additionally, AHEI-2010 considers moderate alcohol intake (Male: 0.5–2.0 drinks/day; Female: 0.5–1.5 drinks/day) as part of a healthful dietary pattern. This means that individuals with moderate alcohol intake received higher scores than non-drinkers (10 points versus 2.5 points). This method of scoring severely penalized nondrinkers, especially for individuals with diabetes. In this sample, the percentage of individuals with alcohol component scores above 2.5 points (drinkers) was approximately 7.7% for individuals with nondiabetes, 7.9% for individuals with prediabetes, and 1.0% for individuals with diabetes. This is likely to be due to the nutrition education and counseling that is typically provided to individuals diagnosed with diabetes (T2DM). As part of diabetes self-management, the ADA recommends that individuals with diabetes (both type 1 and type 2) reduce or minimize alcohol consumption [31], because alcohol intake (especially on an empty stomach) lowers blood glucose and causes hypoglycemia. In addition, many alcoholic beverages contain added sugars, which can lead to excess calories and elevated triglycerides, increasing the risk of heart disease [31]. In this sample, adults with diabetes seemed to minimize alcohol or not drink it at all, which is consistent with diabetes self-management. This suggests that the HEI-2010 may be a better tool for assessing diet quality than the AHEI-2010 for individuals with T2DM.

The present study found a significant linear trend between HEI-2010 and AHEI-2010 quartiles and some of the health markers (Tables 4 and 5). For example, there was a significant decrease in BMI, WC, and triglycerides with increasing total HEI-2010 and AHEI-2010 scores (Tables 4 and 5). These findings are also clinically important because these health markers are negatively influenced by consuming a healthy diet (i.e., whole grains, fruits, vegetables, nuts and legumes). In clinical practice, individuals (especially for diabetes) are often advised to make dietary changes, and compliance to a healthful dietary pattern can lead to improvement in anthropometric or metabolic outcomes. However, there was no difference in CRP levels across HEI-2010 and AHEI-2010 quartiles, which makes the clinical relevance of this health marker to be less clear. Contrary to the present study, previous studies have shown significant inverse association between CRP levels and dietary patterns [51, 52]. Smidowicz and Regula (2015) conducted a systematic review on the role of diet in reducing inflammation and thereby decreasing the risk of chronic disease [52]. The review focused on the effects of several dietary patterns (i.e., Mediterranean diet, DASH diet, low-fat vs. low-carbohydrate) in relation to inflammatory markers (CRP and IL-6) [52]. Based on the review of the research, the authors concluded that it is difficult to determine which dietary pattern is optimal for reducing inflammation. The relationship between inflammation and diet is complex since inflammatory response is often triggered by the cumulative effect of dietary and other factors [52].

Despite the differences in construction of these indices to assess diet, both the HEI-2010 and AHEI-2010 have similar components of a healthful dietary pattern. Overall, the pillars of a healthy diet include higher intakes of fruits, vegetables, whole grains, nuts, legumes, unsaturated fats (i.e., PUFA), and lower intakes of sodium, sugar (i.e., sugar-sweetened beverages) and red and processed meats [49]. Currently, the DGA 2015 does not recommend adherence to a single diet plan to achieve healthy eating patterns, but recommends instead that individuals consume specific food groups that are healthful [50]. Similarly, the ADA recommends that individuals with diabetes consume from various food groups that are high in fiber (i.e., whole grains, vegetables) and avoid foods and/or beverages that contain added sugars to meet metabolic goals such as glucose, HbA1c, lipid levels, and blood pressure [31].

### Strengths and limitations

Strengths of this study include the use of a large, nationally representative sample of U.S. adults with reliable estimates of dietary intake. Therefore, the findings are generalizable and have implications for the development of effective policies to improve health and/or disease outcomes. NHANES is the only national survey that currently provides complete dietary intake through utilizing the AMPM to screen 24-h dietary recalls that are valid and reliable. NHANES has a long history of collecting nutrition data (since the 1960s) and continues to incorporate improvements to refine their dietary methodology.

However, this study also has some limitations: First, NHANES is a cross-sectional study design and therefore, the results cannot support causal inferences about the relationships between HEI-2010 and AHEI-2010 and diabetes status. Second, this study used a single 24-h dietary recall to calculate the HEI-2010 and AHEI-2010 scores, which may not reflect individuals’ habitual or usual intake. In addition, the 24-h recall may be subject to measurement error because it relies on participants’ ability to recall and accurately self-report dietary intake, which may lead to under- or over- reporting. Lastly, NHANES does not explicitly collect information on the type of diabetes (i.e., T1DM or gestational), which may lead to misclassification. However, this study used the available information in NHANES to construct a diabetes classification variable based on using a combination of self-reported and laboratory measured attributes to minimize misclassification.

## Conclusion

In conclusion, HEI-2010 and AHEI-2010 were used as predictors of T2DM, and neither was significant, either alone or in combination with sociodemographic characteristics, health markers, and lifestyle behaviors. However, there were some significant differences in the means of the sub-component HEI-2010 and AHEI-2010 scores by diabetes status. In addition, there were significant positive relationships between the HEI-2010 and AHEI-2010 scores and health markers. Individuals with higher total HEI-2010 and AHEI-2010 scores had better health marker values compared to those with lower diet quality scores. Although total HEI-2010 and AHEI-2010 were not significant predictors of T2DM as expected, the role of diet should not be dismissed as a potential factor in the development of T2DM. There are factors that point to a role of diet in the development of T2DM: the significant differences in means of health markers across HEI-2010 and AHEI-2010 scores, and the significant differences in means of health markers (i.e., BMI, WC, total cholesterol, HDL, LDL, TG, insulin, blood pressure, comorbidity score) by diabetes status. These findings indicate that diet has some influence on T2DM development, leading to the conclusion that better tools are needed to assess dietary intake in persons with diabetes and to better understand the role of diet in T2DM risk.

The main finding of the present study is that diet alone did not have strong predictive ability with respect to T2DM. Neither the HEI-2010 nor the AHEI-2010 performed better than sociodemographics alone as predictors of T2DM. Some sociodemographic characteristics are likely to be associated with genetic differences. This study was not able to assess the impact of genetics, but there has been some recent research investigating the role of genetic factors in the development of T2DM, but this area of inquiry is still in its early stages [53, 54]. In addition, the HEI-2010 and AHEI-2010 were not specifically designed as tools to assess dietary quality in diabetics. Future research is needed to develop an index based on relevant dietary components that contribute to T2DM. This will provide better utility for dietary assessment in adults with diabetes in clinical and community settings.

## Additional file

Additional file 1: **Table S1.** Association between total HEI-2010 score and Diabetes Status in adults (Age ≥ 20, *n* = 3632) using Multinomial Logistic Regression. **Table S2.** Association between total AHEI-2010 score and Diabetes Status in U.S. adults (Age ≥ 20, *n* = 3617) using Multinomial Logistic Regression. (DOCX 25 kb)

## Abbreviations

AHEI-2010: Alternate Healthy Eating Index 2010
BP: Blood Pressure
HDL-C: High density Lipoprotein Cholesterol
HEI-2010: Healthy Eating Index 2010
LDL-C: Low density Lipoprotein Cholesterol
NHANES: National Health and Nutrition Examination Survey
T2DM: Type 2 diabetes mellitus
WC: Waist Circumference

## Footnotes

1 Personal communication with Dr. Joseph Goldman, who is a biostatistician at Food Surveys Research Group, Agricultural Research Service, USDA. 10,300 Baltimore Ave. Bldg. 005, Room 102, BARC-West Beltsville, MD 20705
