# Supplementary material for: Healthy eating index versus alternate healthy index in relation to diabetes status and health markers in U.S. adults: NHANES 2007–2010
Source: Nutr J. 2019 Apr 17;18:26. doi: 10.1186/s12937-019-0450-6 (PMC6471947; doi:10.1186/s12937-019-0450-6)
Supplement: Supplementary file 1 — Table S1. Association between total HEI-2010 score and Diabetes Status in adults (Age ≥ 20, n = 3632) using Multinomial Logistic Regression. Table S2. Association between total AHEI-2010 score and Diabetes Status in U.S. adults (Age ≥ 20, n = 3617) using Multinomial Logistic Regression. (DOCX 25 kb) [file 12937_2019_450_MOESM1_ESM.docx]

**Table S1. Association between total HEI-2010 score and Diabetes Status in adults (Age ≥ 20, n = 3,632) using Multinomial Logistic Regression**

|  | **Prediabetes vs. Non-diabetes** | **Diabetes vs. Non-diabetes** | **Analysis of Effects** | |
| --- | --- | --- | --- | --- |
| **Predictors** | **OR [95% CI]** | **OR [95% CI]** | **Wald Chi-Sq** | **Pr > Chi-sq** |
| Age | 1.10 [1.01, 1.11] | 1.30 [1.20, 1.40] | 55.6 | <0.0001 |
| Age squared | 0.99 [0.99, 1.00] | 1.00 [1.00, 1.00] | 30.3 | <0.0001 |
| Sex |  |  | 27.3 | <0.0001 |
| Male | 1.84 [1.43, 2.40] | 2.21 [1.50, 3.30] |  |  |
| Female | — | — | — | — |
| Ethnicity |  |  | 28.8 | <0.0001 |
| Mexican American | 1.40 [0.98, 1.90] | 2.51 [1.24, 3.90] |  |  |
| Non-Hispanic White | — | — |  |  |
| Non-Hispanic Black | 1.30 [0.93, 1.73] | 2.40 [1.54, 3.70] |  |  |
| Other | 1.20 [0.80, 1.74] | 2.04 [1.20, 3.50] |  |  |
| Self-reported Health |  |  | 45.9 | <0.0001 |
| Excellent | 0.80 [0.60, 1.20] | 0.80 [0.50, 1.30] |  |  |
| Very Good | — | — | — | — |
| Good | 1.30 [1.00, 1.63] | 2.00 [1.20, 3.34] |  |  |
| Fair | 1.20 [0.83, 1.74] | 3.70 [2.14, 6.30] |  |  |
| Poor | 1.10 [0.54, 2.20] | 3.14 [1.40, 7.30] |  |  |
| Smoking Status |  |  | 1.95 | 0.3776 |
| No | — | — | — | — |
| Yes | 1.12 [0.90, 1.50] | 0.90 [0.64, 1.30] |  |  |
| Physical Activity |  |  | 3.80 | 0.1522 |
| No | — | — | — | — |
| Yes | 1.20 [0.98, 1.40] | 0.95 [0.70, 1.35] |  |  |
| Poverty-to-Income Ratio | 0.92 [0.90, 1.00] | 0.92 [0.82, 1.03] | 5.30 | 0.0720 |
| Comorbidity score | 1.10 [1.00, 1.13] | 1.30 [1.20, 1.40] | 35.4 | <0.0001 |
| WC (cm) | 1.03 [1.02, 1.04] | 1.10 [1.04, 1.10] | 89.3 | <0.0001 |
| Total HEI-2010 score | 0.99 [0.98, 1.00] | 1.00 [0.98, 1.01] | 2.9 | 0.2390 |

Baseline outcome category = non-diabetes. Reference categories for categorical predictors are sex (female), ethnicity (non-Hispanic White), self-reported health (very good), smoking status (nonsmoker), physical activity (none).

^*^P-value for the odds of having diabetes and prediabetes compared to nondiabetes (reference group) < 0.05.

Weighted model: Wald- F (32,1) = 34.97, p = 0.1332; goodness-of-fit chi-squared = 16.846, p = 0.396.

*Note*: Included a quadratic term for age as a continuous variable to apply polynomial functions and smoothing splines to test the logistic model is truly linear in the logit. This method was suggested by Hosmer and Lemeshow (2000) and Heeringa, West, and Berglund (2010).

Abbreviations: HEI-2010, Healthy Eating Index 2010; SE, standard error; OR, Odds Ratio; CI, Confidence Interval; WC, Waist Circumference.

**Table S2. Association between total AHEI-2010 score and Diabetes Status in U.S. adults (Age ≥ 20, n = 3,617) using Multinomial Logistic Regression**

|  | **Prediabetes vs. Non-diabetes** | **Diabetes vs. Non-diabetes** | **Analysis of Effects** | |
| --- | --- | --- | --- | --- |
| **Predictors** | **OR [95% CI]** | **OR [95% CI]** | **Wald Chi-Sq** | **Pr > Chi-sq** |
| Age | 1.10 [1.01, 1.11] | 1.30 [1.21, 1.40] | 55.1 | <0.0001 |
| Age squared | 1.00 [1.00, 1.00] | 1.00 [1.00, 1.00] | 30.7 | <0.0001 |
| Sex |  |  | 25.8 | <0.0001 |
| Male | 1.80 [1.40, 2.30] | 2.40 [1.60, 3.62] |  |  |
| Female | — |  | — | — |
| Race/Ethnicity |  |  | 28.8 | <0.0001 |
| Mexican American | 1.40 [1.02, 1.91] | 2.70 [1.72, 4.11] |  |  |
| Non-Hispanic White | — | — | — | — |
| Non-Hispanic Black | 1.30 [0.95, 1.80] | 2.34 [1.52, 3.60] |  |  |
| Other | 1.20 [0.82, 1.80] | 2.02 [1.20, 3.42] |  |  |
| Self-reported Health |  |  | 44.9 | <0.0001 |
| Excellent | 0.80 [0.60, 1.10] | 0.80 [0.50, 1.30] |  |  |
| Very Good | — | — | — | — |
| Good | 1.30 [1.01, 1.70] | 1.97 [1.20, 3.31] |  |  |
| Fair | 1.21 [0.83, 1.80] | 3.55 [2.06, 6.10] |  |  |
| Poor | 1.10 [0.55, 2.22] | 3.00 [1.31, 6.83] |  |  |
| Smoking Status |  |  | 2.50 | 0.2887 |
| Yes | 1.13 [0.90, 1.50] | 0.90 [0.62, 1.30] |  |  |
| No | — | — | — | — |
| Physical Activity |  |  | 2.98 | 0.2251 |
| Yes | 1.14 [0.96, 1.40] | 0.96 [0.68, 1.40] |  |  |
| No | — | — | — | — |
| Poverty-to-Income Ratio | 0.92 [0.85, 0.99] | 0.93 [0.84, 1.04] | 5.98 | 0.0504 |
| Comorbidity score | 1.05 [0.98, 1.14] | 1.30 [1.20, 1.40] | 33.6 | <0.0001 |
| WC (cm) | 1.03 [1.02, 1.04] | 1.06 [1.05, 1.07] | 93.3 | <0.0001 |
| Total AHEI-2010 score | 1.00 [0.99, 1.01] | 1.00 [0.99, 1.01] | 0.21 | 0.9014 |
| Calories (kcal) | 1.00 [0.99, 1.00] | 1.00 [0.99, 1.00] | 6.10 | 0.0485 |

Baseline outcome category = non-diabetes. Reference categories for categorical predictors are sex (female), ethnicity (non-Hispanic White), self-reported health (very good), smoking status (nonsmoker), physical activity (none).^*^P-value for the odds of having diabetes and prediabetes compared to nondiabetes (reference group) < 0.05.

Weighted model: Wald- F (32,1) = 16.73, p = 0.1916; goodness-of-fit chi-squared = 14.119, p = 0.590.

*Note*: Included a quadratic term for age as a continuous variable to apply polynomial functions and smoothing splines to test the logistic model is truly linear in the logit. This method was suggested by Hosmer and Lemeshow (2000) and Heeringa, West, and Berglund (2010).

Abbreviations: AHEI-2010, Alternate Healthy Eating Index 2010; SE, standard error; OR, Odds Ratio; CI, Confidence Interval; WC, Waist Circumference.
